# Hepatitis B und C: Mechanismen der virusinduzierten Leberpathogenese und Tumorentstehung

**DOI:** 10.1007/s00103-021-03482-y

**Published:** 2022-01-11

**Authors:** Mirco Glitscher, Eberhard Hildt, Daniela Bender

**Affiliations:** Abteilung 2/01, Virologie, Paul-Ehrlich-Institut – Bundesinstitut für Impfstoffe und biomedizinische Arzneimittel, Paul-Ehrlich-Str. 51–59, 63226 Langen, Deutschland

**Keywords:** Hepatitis-B-Virus, Hepatitis-C-Virus, HCC, Regulatorische Proteine, Signaltransduktion, Hepatitis B virus, Hepatitis C virus, HCC, Regulatory proteins, Signal transduction

## Abstract

Die Hepatitisviren B und C (HBV, HCV) sind weltweit die relevantesten viralen Auslöser einer chronischen Hepatitis (Leberentzündung). Derzeit leiden weltweit mehr als 250 Mio. Menschen an einer chronischen HBV-Infektion, jährlich versterben 0,8 Mio. an den Folgen. Von einer chronischen HCV-Infektion sind ca. 70 Mio. Menschen betroffen, es versterben ca. 1 Mio. im Jahr. Bisher steht nur für HBV eine zugelassene Impfung zur Verfügung. Chronische Infektionen mit HBV und HCV gehen mit einem erhöhten Risiko für die Entwicklung einer Leberfibrose, einer Leberzirrhose und eines hepatozellulären Karzinoms (HCC) einher.

Diese Übersichtsarbeit beschreibt Mechanismen der HBV- und HCV-assoziierten Pathogenese. Im Vordergrund stehen dabei die Wechselwirkung der chronischen Infektion mit intrazellulären Signaltransduktionswegen, mit einzelnen Stoffwechselwegen, insbesondere dem Lipidmetabolismus, die Fibrose- und Zirrhoseentstehung im Laufe der chronischen Infektion sowie Mechanismen der virusinduzierten HCC-Entstehung.

Trotz großer Fortschritte in der Charakterisierung der viralen Lebenszyklen und der Entwicklung robuster antiviraler Strategien bleiben Herausforderungen bestehen: u. a. die Gewinnung eines noch besseren Verständnisses der Mechanismen, die zur Entwicklung der virusassoziierten Pathogenese beitragen, sowie die Erforschung der Relevanz verschiedener Genotypen für Unterschiede in der Pathogenese.

## Abstract (en)

Worldwide, the hepatitis B and hepatitis C viruses (HBV, HCV) are the most relevant causative viral agents of a chronic hepatitis (inflammation of the liver). At present, more than 250 million people suffer from a chronic HBV infection globally, resulting in 0.8 million deaths per year. A chronic HCV infection accounts for about 70 million cases worldwide, leading to a death toll of about 1 million per year. An approved vaccine is only available against an HBV infection. Both HBV and HCV infections result in a highly increased risk of developing liver fibrosis, cirrhosis, and a hepatocellular carcinoma (HCC).

This review aims to describe mechanisms of the HBV- and HCV-associated pathogenesis. The focus is on the interplay between a chronic infection with intracellular signaling transduction, metabolic pathways with an emphasis on lipid metabolism, the establishment of liver fibrosis and cirrhosis during a chronic infection, and the mechanisms of the onset of a virally induced HCC.

Despite there being great advances in the characterization of viral life cycles and the development of robust antiviral strategies, significant hurdles persist: gaining a better understanding of the mechanisms that drive virus-associated pathogenesis as well as increasing insights regarding different viral genotypes having impacts on alternate pathogeneses.

## Einleitung

Chronische Infektionen mit dem Hepatitis-B-Virus (HBV) und dem Hepatitis-C-Virus (HCV) sind weltweit die relevantesten viralen Auslöser einer chronischen Hepatitis (Leberentzündung).

Trotz der Entwicklung einer sicheren und sehr wirksamen Impfung gegen HBV zählt die chronische HBV-Infektion mit den aus ihr resultierenden Komplikationen zu den 30 häufigsten Todesursachen weltweit. Derzeit leiden nach Schätzungen der Weltgesundheitsorganisation (WHO) ca. 257 Mio. Menschen an einer chronischen Infektion mit HBV und mehr als 0,8 Mio. Menschen sterben jährlich an den Folgen. Die Prävalenz ist in weiten Teilen Südostasiens und Zentralafrikas sehr hoch, in Mitteleuropa hingegen sehr gering. So liegt sie beispielsweise in Deutschland bei unter 1 %, ist aber in den letzten Jahren leicht ansteigend [1, 2].

**Figure 1:**
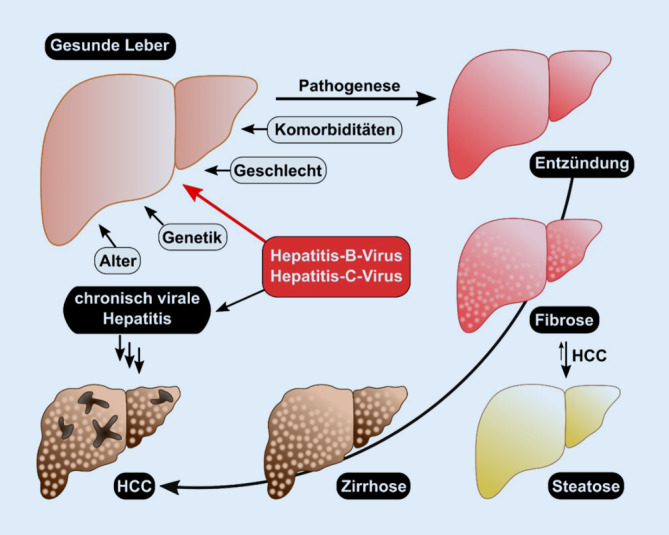

Weltweit leiden derzeit ca. 70 Mio. Menschen an einer chronischen Infektion mit HCV. Im Unterschied zu HBV ist gegen HCV derzeit noch keine Impfung verfügbar. Die Persistenz der HCV-Infektion führt, wie auch die der HBV-Infektion, zu einer chronischen Infektion, die zu einer Zirrhose und einem hepatozellulären Karzinom (HCC) voranschreiten kann (Abb. 1). Mit der Entwicklung der direkt antiviral wirksamen Medikamente (engl.: „direct acting antivirals“, DAA) wurde eine Therapiemöglichkeit gegen HCV eröffnet, die weltweit aber nur begrenzt verfügbar ist. Einen zugelassenen HCV-Impfstoff gibt es derzeit noch nicht [1, 3].

Die molekulare Virologie von HCV und HBV wird im Beitrag von Bender et al. in diesem Themenheft beschrieben, sodass hier nicht darauf eingegangen wird. Die vorliegende Übersichtsarbeit beschreibt Aspekte der HBV- und HCV-assoziierten Pathogenese und einige zugrunde liegende Mechanismen.

## Destruktion und Regeneration der Leber

Der von Hesiod im 8. Jahrhundert vor Christus überlieferte Mythos von Prometheus zeigt, wie alt das Wissen um die Regenerationsfähigkeit der Leber ist. Dieses Organ hat die Eigenschaft, sein eigenes Gewebe nach einer Schädigung durch Toxine, Viren oder Verletzungen zu regenerieren. Allerdings ist das Auftreten von Fibrose und Zirrhose, also das Ersetzen funktionellen Lebergewebes durch unfunktionales Bindegewebe, in vielen Fällen einer chronischen HCV- wie auch HBV-Infektion zu beobachten, was im Kontrast mit der klassischen Leberregeneration steht. Die chronische Infektion ist häufig durch eine insuffiziente (zelluläre) Immunantwort gekennzeichnet, die nur teilweise zu einer Zerstörung HBV- bzw. HCV-positiver Hepatozyten, aber nicht zu deren vollständiger Eliminierung führt [4]. Es kommt zunächst zu einem Prozess abwechselnder Destruktion und Regeneration. Über einen längeren Zeitraum verschiebt sich jedoch das Gleichgewicht und funktionelles Lebergewebe wird durch eingewanderte Fibroblasten und dann durch Bindegewebe ersetzt. Ein entscheidender Faktor für die Kontrolle der Leberregeneration sind insulinabhängige Prozesse [5], die sowohl bei HBV- als auch bei HCV-Infektionen gestört werden, allerdings auf molekularer Ebene auf völlig verschiedenen Mechanismen beruhen.

## HCV bedingt eine Erhöhung des intrazellulären Spiegels von reaktiven Sauerstoffspezies (ROS)

Die HCV-Strukturproteine Core, E1 und E2 sowie die Nichtstrukturproteine NS3, NS4B und NS5A tragen auf unterschiedlichen Wegen zur Generierung von reaktiven Sauerstoffspezies („reactive oxygen species“, ROS) in Hepatozyten bei [6]. Umgekehrt gibt es in den Zellen eine Vielzahl von Mechanismen, welche die Entgiftung von ROS vermitteln können. Eine zentrale Rolle spielt dabei der Transkriptionsfaktor Nrf2 („nuclear factor erythroid 2‑related factor 2“), der in die Regulation der Expression einer Vielzahl zytoprotektiver Gene involviert ist. In inaktivem Zustand liegt Nrf2 komplexiert mit seinem Inhibitor Keap1 (Kelch-like Ech-associated Protein 1) vor. Bei einer Aktivierung von Nrf2, beispielsweise durch Elektrophile oder durch einen erhöhten ROS-Spiegel, kommt es zu einer Dissoziation von Nrf2 und Keap1. Nrf2 wandert in den Zellkern, wo er als Heterodimer mit kleinen Maf-Proteinen („small musculo-aponeurotic fibrosarcoma“, sMaf) an das sog. ARE („antioxidant response element“) bindet, das sich in der Promotorregion vieler zytoprotektiver Gene befindet [7, 8].

In HCV-positiven Zellen kommt es zu einer Hemmung der Induktion Nrf2-ARE-abhängiger Gene. Ursächlich hierfür ist die Verlagerung von sMaf-Proteinen aus dem Zellkern an die Außenseite des endoplasmatischen Retikulums (ER), wo sie mit NS3 interagieren. Dies wiederum bewirkt, dass „aktiviertes“ Nrf2, das nicht mehr an Keap1 gebunden ist, nicht in den Zellkern einwandern und dort auch nicht als Heterodimer mit sMaf die Expression zytoprotektiver Gene anschalten kann. Durch diese Genexpressionshemmung wird die Detoxifizierung von ROS verhindert und es kommt zu einem erhöhten ROS-Spiegel. Der in HCV-positiven Zellen vorliegende erhöhte ROS-Spiegel hat verschiedene Implikationen [9–11].

Zum einen benötigt HCV einen erhöhten ROS-Spiegel für den Ablauf des viralen Replikationszyklus. Hierbei kommt es zu einer ROS-abhängigen Induktion der Autophagie, die wiederum relevant ist für die Freisetzung von HCV durch exosomale Vesikel. Verschiedene Mechanismen sichern den Erhalt eines erhöhten ROS-Spiegels in HCV-positiven Zellen. Der erhöhte ROS-Spiegel bewirkt weiterhin eine Phosphorylierung des Proteins p62. P‑p62 wiederum bindet statt Nrf2 an Keap1 und bedingt so die Freisetzung („Aktivierung“) von Nrf2 aus dem Komplex mit Keap1. Dies würde nun die Aktivierung von Nrf2-ARE-abhängigen Genen bedingen und somit den für die HCV-Replikation essenziellen Prozess der ROS-abhängigen Induktion der Autophagie beenden und daher den Lebenszyklus zum Stillstand bringen. Der ungewöhnliche Prozess der Hemmung der Nrf2-ARE-abhängigen Genexpression durch die Translokation von sMaf an die ER-Außenseite verhindert jedoch das Einwandern von freigesetztem („aktiviertem“) Nrf2 in den Zellkern und somit die Expression zytoprotektiver Gene. Dies wiederum verhindert die ROS-Detoxifizierung [11, 12].

## Pathologische Relevanz des erhöhten ROS-Spiegels auf die Leberregeneration bei HCV-Infektion

Eine physiologische Konsequenz des erhöhten ROS-Spiegels ist der Einfluss auf die genomische Integrität der Wirtszelle. Aber auch auf die Integrität des Virusgenoms hat er Auswirkungen, was zur Entstehung von Virusvarianten („Quasispezies“) beitragen könnte. Der ROS-abhängigen Induktion von Mutationen im Wirtsgenom wird eine wesentliche Rolle bei der Tumorgenese durch die mutagene Wirkung zugeschrieben.

Daneben ist der erhöhte ROS-Spiegel von großer Bedeutung für die sog. Insulinresistenz in Hepatozyten. Insulinabhängige Signalwege spielen eine wesentliche Rolle bei der Kontrolle der Leberregeneration. Über den Insulinrezeptor (IR) kommt es zur Aktivierung von proliferativen und antiapoptotischen Signalwegen. Der IR gehört zur Familie der Tyrosinkinasen. Bei Induktion proliferativer Signale durch Insulin kommt es zunächst zu einer Bindung von tyrosin-phosphoryliertem IRS1/2 (Insulinrezeptorsubstrat 1/2) an den IR. Dies führt über eine Aktivierung von PI3K (Phosphoinositid-3-Kinasen) und AKT/PKB (Proteinkinase B) einerseits zur Aktivierung des S6K/p70-Kinasekomplexes und andererseits zu einer Hemmung des proapoptotischen Proteins BAD und der GSK‑3 Beta (Glykogensynthase-Kinase 3 Beta). Letztlich bedingt dies eine Aktivierung von proliferativen und antiapoptotischen Signalen.

Bei erhöhten intrazellulären ROS-Spiegeln kommt es jedoch zu einer von JNK1/2 (Jun-Kinase 1/2) abhängigen Serin/Threonin-(Ser/Thr‑)Phosphorylierung von IRS1/2. Ser/Thr-phosphoryliertes IRS1 ist nicht in der Lage, an IR zu binden, und verhindert somit das Weiterleiten von proliferativen/antiapoptotischen Signalen nach Insulinbindung an IR [13].

Neben dieser funktionellen Hemmung des IR-Signalwegs kann es in HCV-positiven Zellen, wie für Genotyp 3 auch beschrieben, zu einer verminderten Expression der Phosphatase PTEN kommen. Die verminderte Expression von PTEN scheint relevant für den HCV-Lebenszyklus zu sein, da eine Überexpression von PTEN in HCV-replizierenden Zellen zu einer verminderten Freisetzung von HCV führt [14].

Epidemiologische Daten weisen auch darauf hin, dass diese IR-assoziierten Mechanismen zu einer Störung des Glukosemetabolismus führen könnten, da eine chronische HCV-Infektion mit einem erhöhten Risiko für die Entwicklung eines Diabetes Typ 2 (T2DM) einhergeht [15, 16]. Neben der Hemmung der insulinrezeptorabhängigen Signaltransduktion trägt hierzu auch eine verminderte Expression der Glukosetransporter 1 und 2 bei. Dazu kommt eine verstärkte Bildung von TNF‑α (Tumornekrosefaktor-Alpha), was eine Hemmung der Glukosetransporter 1 und 2 auslöst [17, 18]. Neben der Ser/Thr-Phosphorylierung von IRS1/2 in HCV-replizierenden Zellen durch JNK (Jun-Kinase) werden auch eine core-abhängige verstärkte Ubiquitinierung und nachfolgende Degradation von IRS1/2 beschrieben [18].

## Reduktion der Menge an Insulinrezeptoren auf der Oberfläche HBV-replizierender Zellen

Ein grundsätzlich anderer Mechanismus wird für HBV diskutiert. Es gibt vielfältige übereinstimmende Berichte, dass HBV beispielsweise durch eine „Überladung“ des ER bei Retention des Oberflächenproteins LHBs („large hepatitis B virus surface protein“) oder durch Wechselwirkung des multifunktionalen Hepatitis-B-X-Proteins (HBx) mit mitochondrialen Strukturen zur Generierung von ROS führen kann, wie auch durch die Prozesse der chronischen Inflammation [19]. Dagegen sind die Publikationen zum Einfluss von HBV auf die Nrf2-ARE-abhängige Genexpression konfliktär. Die beschriebenen unterschiedlichen Effekte können evtl. durch Unterschiede zwischen den jeweils untersuchten Genotypen bedingt sein. So wurde beispielsweise für Genotyp D berichtet, dass es in HBV-exprimierenden Zellen zu einer Kinase(c)-Raf-abhängigen Aktivierung von Nrf2-ARE-abhängigen Promotoren kommt und somit zu einer verstärkten Expression zytoprotektiver Gene. Ursächlich dafür sind durch das regulatorische Protein HBx und die Aktivatoren der LHBs-codierenden Genregion PreS2 deregulierte Signalwege. Unklar ist aber, inwieweit die beobachtete Aktivierung Nrf2-ARE-abhängig induzierter zytoprotektiver Gene in vivo tatsächlich ausreicht, um die im Zuge des Infektionsprozesses gebildeten ROS zu entgiften, und inwieweit dies dann zu einer Insulinresistenz in HBV-positiven Zellen beiträgt [20–22].

Interessanterweise konnte in vitro und in vivo jedoch beobachtet werden, dass es in HBV-produzierenden Zellen/Geweben zu einer verstärkten Expression von IR und zu einer erhöhten Menge im Vergleich zu den HBV-negativen Kontrollen kommt. Ein wesentlicher Faktor bei der HBV-abhängigen Induktion der IR-Expression ist die HBV-abhängige Aktivierung von Nrf2 durch HBx und die PreS2-Aktivatoren. Allerdings ist die Menge an IR an der Zelloberfläche in den HBV-positiven Zellen gegenüber den Kontrollen vermindert, sodass es zu einem funktionellen Abkoppeln von Insulinsignalen kommt. Die intrazelluläre Retention von IR in HBV-positiven Zellen wird durch eine deutlich erhöhte Menge an Alpha-Taxilin, einem syntaxin4-bindenden Protein, bedingt. Hemmung der Alpha-Taxilin-Expression führt zu einem deutlichen Anstieg der Menge von IR an der Zelloberfläche von HBV-positiven Zellen [23, 24]. Inwieweit diese Mechanismen auch bei chronisch HBV-infizierten Patienten zur T2DM beitragen, ist unklar. Eine koreanische Studie beschreibt, dass eine chronische HBV-Infektion mit Insulinresistenz assoziiert ist, aber hierzu sind weitere Studien erforderlich [25, 26].

HBV und HCV bedingen also durch molekular vollkommen unterschiedliche Mechanismen ein Abkoppeln der Zelle von proliferativen Insulinsignalwegen, was ein wesentlicher Faktor für die Entstehung von Fibrose und Zirrhose ist.

## HBV und HCV unterscheiden sich fundamental in ihrem Einfluss auf den Lipidmetabolismus

Der HCV-Lebenszyklus ist an verschiedenen Stellen eng mit dem Lipidmetabolismus verbunden [27, 28]. Dabei beeinflusst HCV Schlüsselschritte des Lipidmetabolismus, des Lipidtransports und der Lipidaufnahme. Die HCV-Replikation geht mit einem intensiven Umbau von ER-Strukturen einher, die zusammen mit der Ausbildung von Lipidtröpfchen (engl.: „lipid droplets“, LD) zur Entstehung des sog. Membranous Web führen, dem Ort der viralen RNA-Vermehrung. Ein Charakteristikum von HCV ist die Ausbildung zytosolischer Doppelmembranvesikel (DMV), im Unterschied zu anderen Flaviviren, wo die Invagination in das ER-Lumen erfolgt. Neben diesen strukturellen Veränderungen kommt es zu einer deutlichen Änderung im Lipidmetabolismus. Bei der HCV-Infektion kommt es zu einer Translokation von PI4KIIIa (Phosphatidylinositol-4-Kinase) vom Golgi-Komplex und der Plasmamembran zum ER, vermittelt durch eine Interaktion mit NS5A. Am ER kommt es dann zu einer verstärkten Bildung von PI4P (Phosphatidylinositol-4-Phosphat). Eine Konsequenz daraus ist die Anreicherung von Sphingolipiden und Cholesterin im Bereich der DMV und die Bildung von sog. Lipid Rafts („Lipidflößen“). Dabei akkumulieren Glycosphingolipide an PI4P-angereicherten Membranen indem sie mit dem Lipidtransferprotein FAPP2 interagieren [29, 30].

Die HCV-Morphogenese hängt vom Vorhandensein der LDs ab. Diese bestehen aus einem „Kern“ aus Triglyceriden und Cholesterolestern, der von einem Phospholipidmonolayer umgeben ist. Sie befinden sich in HCV-replizierenden Zellen überwiegend im Membranous Web [27–30]. NS5A transportiert die neu gebildeten Genome von den Replikonkomplexen zu den Core-Proteinen, die sich an der Oberfläche der LD befinden, wo die Verpackung in Kapside erfolgt. Eine wesentliche Rolle für den gerichteten Transport des NS5A-RNA-Komplexes spielt TIP47 (Tail-interacting 47-kDa Protein), das an NS5A binden kann [31–33]. Core und NS5A binden DGAT1 (Diacylglycerol-O-Acyltransferase 1) am ER und assoziieren mit DGAT1-abhängig generierten LD. Im Zuge der HCV-Infektion kommt es zu einer erhöhten Menge an LD. Insbesondere bei HCV-Genotyp 3 tritt eine Verfettung (Steatose) der Leber auf. Dabei sind eine verminderte Expression von PTEN zu beobachten und ein erhöhter Anteil an Cholesterinestern in den LD. In diesem Zusammenhang wurde beschrieben, dass Core die Expression von SREBP-1c (Sterol Regulatory Element-binding Protein-1c) induziert – ein wesentlicher Transkriptionsfaktor bei der Kontrolle der Synthese von Triglyceriden, Fettsäuren und Phospholipiden. Auch die Interaktion von NS5A mit Apolipoprotein A1 und A2 spielt eine wesentliche Rolle für den Einfluss von HCV auf den Gehalt von Triglyceriden in Hepatozyten. Weiterhin interagiert Core mit der ATGL (Adipozyten-Triglycerid-Lipase) und inhibiert dadurch deren hydrolytische Funktion [34–36].

Wie oben beschrieben induziert HCV metabolische Deregulation und geht mit Insulinresistenz, T2DM, Steatose und metabolischem Syndrom (MetS) einher. Dabei liegt die Prävalenz des MetS bei HCV-Patienten zwischen 13 % und 31 %, wobei die Prävalenz mit dem Alter zunimmt [16, 36].

Bei HBV-Patienten hingegen gibt es keinen Hinweis auf ein erhöhtes Risiko für das Auftreten des MetS. Vielmehr gibt es Berichte, die beschreiben, dass eine chronische HBV-Infektion das Risiko, MetS zu entwickeln, vermindert [16].

Für die Entwicklung einer nichtalkoholischen Fettleberkrankheit (NAFLD) besteht bei chronischer HCV-Infektion eine Prävalenz von 55 %, wobei hier insbesondere Infektionen mit Genotyp 3 ursächlich sind. Dagegen gibt es keinen Hinweis auf eine erhöhte Prävalenz von Fettleber bei chronischen HBV-Patienten. Einzelne Studien weisen sogar auf eine niedrigere Prävalenz im Vergleich zur Kontrollgruppe hin, insbesondere bei älteren Patienten. Interessanterweise kommt es bei HBV/HCV-Doppelinfektionen zu einer vergleichbaren Prävalenz der Steatose wie bei einer HCV-Monoinfektion [16, 37].

## Integration von HBV-DNA

Obgleich es im Zuge der HBV-Replikation zu reverser Transkription kommt, ist – im Unterschied zu Retroviren – die Integration in das Wirtsgenom kein essenzieller Schritt des viralen Lebenszyklus. Ausgehend von natürlich auftretenden Integraten wird kein infektiöses Virus gebildet. Nach der reversen Transkription der sog. Pregenomic RNA (pgRNA) entsteht eine partiell doppelsträngige Relaxed Circular DNA (rcDNA), aus der dann Covalently Closed Circular DNA (cccDNA) gebildet werden kann. Von dieser cccDNA ausgehend kann die HBV-Replikation weiter erfolgen. Daneben entsteht bis zu ca. 10 % doppelsträngige lineare DNA (dslDNA; [38]). Auch in Virionen und nackten Kapsiden kann dslDNA nachgewiesen werden. Die Integration der dslDNA in das Wirtsgenom erfolgt in ca. 1 von 10^5^ bis 10^6^ infizierten Hepatozyten. Sie kann bereits in einer frühen Phase der Infektion stattfinden und durch verschiedene Mechanismen zur HCC-Entstehung beitragen. Dazu gehören (i) die Vermittlung chromosomaler Instabilität, (ii) die insertionelle Mutagenese von Tumorsuppressoren und Protoonkogenen und (iii) die Bildung mutierter Proteine ausgehend von integrierter HBV-DNA, wie beispielsweise C‑terminal-verkürzte Oberflächenproteine wie etwa MHBst („medium hepatitis B virus surface protein“; [39, 40]).

Während in Nicht-HCC-Patienten die HBV-Integration zufällig über das ganze Genom der Wirtszelle erfolgt, wird in CHB-HCC-Patienten vermehrt eine Häufung von Integration in fragilen DNA-Bereichen des Wirtsgenoms beobachtet. Dazu zählen repetitive Regionen, insbesondere LINEs („long interspersed nuclear elements“), CpG-Inseln und Telomere. In einer Vielzahl HBV-assoziierter HCC können auch chromosomale Aberrationen beobachtet werden. Weiterhin ist zu beobachten, dass in HBV-HCC-Geweben eine größere Anzahl an Integraten zu finden ist als im umliegenden HBV-positiven Nicht-HCC-Gewebe. In 10–15 % der HBV-assoziierten HCC ist die Integration der Enhancer-II/Core-HBV-Promotorregion insbesondere im Bereich des TERT-Gens zu beobachten, was zu einer verstärkten Expression führen kann und somit den Transformationsprozess fördern kann [41, 42].

## Deregulation intrazellulärer Signaltransduktionswege durch HBV-Proteine

### HBx

**Figure 2:**
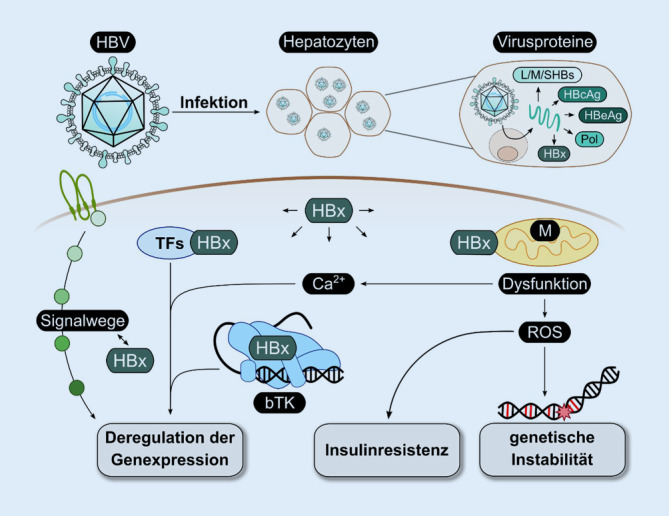

HBx werden eine Vielzahl von Funktionen hinsichtlich des HBV-Lebenszyklus und der virusassoziierten Pathogenese zugeschrieben (Abb. 2). Bei der Interpretation dieser Daten ist jedoch zu bedenken, dass HBx im Verlauf der natürlichen Infektion nur in sehr geringen Mengen gebildet wird, sodass bisweilen die physiologische Relevanz von Daten, die auf einer sehr starken Überexpression und stöchiometrisch ausgeglichenen Mengenverhältnissen zwischen HBx und Ligand basieren, zu hinterfragen ist. Dies betrifft u. a. auch die Frage der Löslichkeit des überproduzierten HBx unter diesen Bedingungen. So ist das Vorliegen von HBx-Aggregaten in unlöslicher Form zu beobachten.

Ein Teil der beschriebenen Effekte von HBx auf die Transkription zellulärer und viraler Gene basiert auf der Interaktion mit Transkriptionsfaktoren (ATF/CREB, ATF3, c/EBP ETS, SMAD4, OCT1), auf Komponenten der basalen Transkriptionsmaschinerie sowie auf epigenetischen Modifikationen. Dies führt zur Induktion bzw. Hemmung der Expression. So wird eine Bindung von HBx an CREB-regulierte Promotoren beschrieben. Durch die verstärkte Rekrutierung von CBP/p300 und Hemmung der CREB-Inaktivierung kommt es zu einer Aktivierung dieser Promotoren [43, 44].

Ein weiterer HBx-abhängiger Mechanismus basiert auf der Degradation von SUZ12, einer Komponente von PRC2 (Polycomb Repressive Complex 2), das die Histonmethylierung beeinflusst. Eine HBx-abhängige Verminderung der Transkription durch eine verstärkte Aktivität der DNA-Methyltransferase (DNMT) kann durch Induktion von DNMT1, DNMT3A1 und DNMT3A2 erzielt werden. Für die Unterstützung der HBV-Replikation spielt der HBx-abhängige Abbau der DNA-Reparaturproteine Smc5/6 eine wesentliche Rolle. HBx verknüpft Smc5/6 mit der E3-Ubiquitin-Ligase, was den Abbau von Smc5/6 fördert und somit die strukturelle Veränderung der cccDNA und damit deren Transkription ermöglicht [45].

Neben der Aktivierung von Signaltransduktionswegen, die als Tumor-Promotor-Pathways angesehen werden können, beeinflusst HBx eine Vielzahl weiterer Mechanismen, die relevant für den Prozess der HCC-Entstehung sein können. Dazu zählt auch die Aktivierung des Beta-Catenin-Signalwegs durch HBx. Hierbei kann HBx die vom Glycoprotein C1q abhängige Aktivierung des Beta-Catenin-Signalwegs insbesondere in HPC (Hepatic Progenitor Cells) verstärken [43, 44].

Eine wesentliche Rolle bei der HBV-assoziierten Pathogenese wird auch der Interaktion von HBx mit dem Tumorsuppressorprotein p53 zugeschrieben, was zur funktionellen Inaktivierung von p53 führen kann. Darüber hinaus wird eine Delokalisation im Zytoplasma von p53 durch die Interaktion mit HBx beschrieben. Hierbei stellt sich, wie schon oben beschrieben, die Frage nach den stöchiometrischen Verhältnissen: Ist die relativ kleine Menge an HBx ausreichend, um p53 effizient zu inaktivieren? Ebenso wird die Frage der Lokalisation von HBx konfliktär beschrieben [46].

Weitere Funktionswege von HBx basieren auf einer Deregulation von Signaltransduktionskaskaden, wie beispielsweise dem c‑Raf-MEK-Erk-Kinase-Signalweg. Dadurch kann HBx eine tumorpromotoranaloge Wirkung ausüben [43, 44, 47].

Das HBV-assoziierte HCC tritt deutlich häufiger bei Männern auf. Hierbei ist eine Korrelation zwischen der Aktivität des Androgenrezeptorsignalwegs und dem HCC-Risiko bei Männern beschrieben. HBx kann dabei die androgenabhängige AR-(Androgenrezeptor‑)Aktivität in vitro und in vivo verstärken, indem es das Enzym c‑Src aktiviert, GSK-3-Beta-Kinasen inhibiert und die Phosphorylierung von AR stimuliert. Dies bewirkt die Translokation von AR-Dimeren in den Zellkern und somit die Expression AR-abhängig regulierter Gene ([48]; Abb. 2).

### PreS2-Aktivatoren

Den Oberflächenproteinen von HBV wird teilweise ebenfalls eine regulatorische Funktion zugeschrieben. Dabei werden verschiedene Mechanismen diskutiert. Eine sehr frühe Beobachtung war, dass es bei selektiver Überexpression von LHBs zu einer intrazellulären Retention und Akkumulation im ER kommt, was letztlich zu einer starken morphologischen Veränderung der Hepatozyten führt, den sog. Milchglashepatozyten („ground glass hepatocytes“; [49]). Durch diese Akkumulation kann ER-Stress entstehen, der eine verstärkte Ca^2+^-Freisetzung ins Zytosol induziert. Die dadurch bedingte mitochondriale Dysfunktion führt zur Entstehung von ROS. Daneben kann es durch Ausbildung langer filamentöser Strukturen zu inflammatorischen Prozessen kommen, die teilweise denen ähneln, die in der Lunge durch Asbestfasern ausgelöst werden können.

Ausgehend von der Charakterisierung integrierter HBV-DNA aus HCC-Geweben konnten C‑terminal-verkürzte Oberflächenproteine identifiziert werden, die eine regulatorische Funktion ausüben [50, 51]. Weitere Untersuchungen zeigten, dass die PreS2-Domäne ausreichend für die Vermittlung der transkriptionellen Aktivatorfunktion ist. Voraussetzung ist die zytoplasmatische Orientierung der PreS2-Domäne, wie im Falle C‑terminal-verkürzter MHBst, aber auch im Falle des kompletten LHBs, das hinsichtlich der PreS1PreS2-Domäne eine duale Membrantopologie aufweist. Die zytoplasmatisch orientierte PreS2-Domäne interagiert mit den klassischen Proteinkinase-C-Isoformen a und b, aktiviert diese und dies wiederum bedingt eine Aktivierung des Raf-MAP-Kinase-Signalwegs [52–54].

Neben C‑terminalen Deletionen im Bereich des S‑Gens führen auch partielle Deletionen im Bereich der PreS1PreS2-Domäne zu geänderten Eigenschaften. PreS2-Deletionen induzieren vom Enzym mTOR abhängige glykolytische Signalwege oder Lipidakkumulationen durch eine Aktivierung von SREBP‑1. Darüber hinaus wird für PreS2-Mutanten eine Interaktion mit JUN Activation Domain-binding Protein 1 (JAB1) beschrieben, die einen Abbau des Cyclin-Dependent-Kinase-(CDK-)Inhibitors p27 und die Hyperphosphorylierung von pRb (Retinoblastom-Protein, Tumorsuppressor) bedingt [55, 56]. Der Einfluss auf den Abbau von p27 und somit auf die Zellzykluskontrolle wurde wiederum auch für nichtmutierte, zytoplasmatisch orientierte PreS2-Domänen beschrieben [57].

## Deregulation intrazellulärer Signaltransduktionswege durch HCV-Proteine

**Figure 3:**
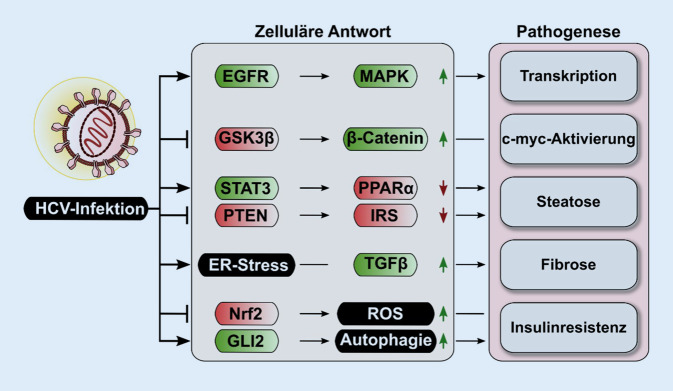

Die Aktivierung proliferativer und antiapoptotischer Signalwege wird für die HCV-Proteine E2, NS2, NS3, NS4A, NS5A und NS5B beschrieben (Abb. 3). Eine wesentliche Rolle bei der HCV-abhängigen Induktion der Zellproliferation spielt NS5B, das an den Tumorsuppressor pRb binden kann und somit dessen proteasomalen Abbau fördert, was u. a. die Freisetzung des ribosomalen Proteins E2F (Elongation Factor 2F) auslöst [58]. Für NS2 wird eine Aktivierung des Cyclin/CDK4-Komplexes beschrieben, was die Expression von Cyclin E induziert [59]. Auch für das HCV-Core-Protein sind Interferenzen mit der Zellzykluskontrolle beschrieben: Dazu gehört eine verstärkte Expression von Cyclin E/CDK2, um im Zellzyklus den Übergang aus der G1-Phase in die S‑Phase zu erleichtern [36, 58]. NS5A kann mit PTEN interagieren und ihn dadurch inaktivieren [60]. NS5A kann c‑Raf binden und aktivieren, was aber nicht mit einer Aktivierung von Erk1/2 einhergehen muss. NS5A weist in seinem N‑terminalen Bereich eine amphipathische Struktur auf, welche die Interaktion mit dem ER vermittelt. Im C‑terminalen Bereich weist NS5A ein Kernlokalisationssignal auf. Im Zuge posttranslationaler proteolytischer Prozessierungen können N‑terminal-verkürzte NS5A-Fragmente entstehen, die dann aufgrund des C‑terminalen Kernlokalisierungssignals (NLS) in den Zellkern einwandern. Da diese Fragmente weiterhin c‑Raf binden können, führt dies zu einem Entzug von c‑Raf aus der klassischen Signalkaskade und somit zu einer Unterbrechung des c‑Raf-MEK-Erk-Signalweges [61, 62].

NS5A aktiviert den Wnt/Beta-Catenin-Pathway durch die Bindung von Beta-Catenin und durch die Induktion der Phosphorylierung von GSK‑3 Beta. Ein weiterer Bindungspartner von NS5A ist p53, wodurch NS5A ein antiapoptotisches Potenzial zugeschrieben wird. NS2 und NS3/4A induzieren eine Delokalisation von p53 aus dem Zellkern ins Zytoplasma und können somit ebenfalls einen antiapoptotischen Effekt ausüben. Auch für das Core-Protein wird eine Interaktion mit p53 beschrieben, allerdings ist dieser Effekt mengenabhängig [36, 58, 60].

NS5A bindet weiterhin an den Transforming-Growth-Factor-(TGF-)Beta-Rezeptor1 und hemmt dadurch TGF-beta-abhängige Signalwege, indem es die Phosphorylierung durch die nukleäre Translokation von SMAD2 und des SMAD3/SMAD4-Heterodimers hemmt. Für das Core-Protein ist ebenfalls eine Interaktion mit dem TGF-Beta-Signalweg beschrieben. Core interagiert direkt mit dem SMAD3 und verhindert dadurch die DNA-Bindung des SMAD3/SMAD4-Heterodimers ([58, 63]; Abb. 3).

## Permanente Inflammation

Persistierende inflammatorische Prozesse tragen ganz wesentlich zur Entstehung und Progression von HCC bei. Mehr als 80 % der HCC entwickeln sich vor dem Hintergrund einer chronischen Inflammation. Bei chronischer HCV-Infektion entwickelt sich das HCC in der Regel vor dem Hintergrund einer Zirrhose. Bei HBV-assoziierten HCC kommt es in 10–20 % der Fälle zur HCC-Entstehung, ohne dass das Stadium der Zirrhose und chronisch entzündlicher Prozesse durchlaufen wird [36, 64]. Im Zuge der Infektion kommt es zur Induktion der angeborenen und der erworbenen Immunantwort. Dabei kommt es zur Eliminierung des Pathogens durch zytolytische und nichtzytolytische Prozesse. Bei nicht erfolgreicher Eliminierung des Pathogens können inflammatorische Stimuli persistieren, was zu einer permanenten Inflammation und gestörten Geweberegeneration führt. Bei einer HBV-Infektion ist der Übergang zur chronischen Hepatitis durch eine Aktivierung der adaptiven Immunantwort, die mit der Gegenwart HBV-spezifischer CD8+-T-Zellen (CD8: Oberflächenmarker) und der Sekretion proinflammatorischer Zytokine einhergeht, gekennzeichnet. Dabei wird die Zerstörung des Lebergewebes nicht mehr vollständig durch regenerative Prozesse funktionell kompensiert. Die Immunpathogenese ist weiter verstärkt durch die Freisetzung von ROS aus den CD8+-T-Zellen und den eingewanderten natürlichen Killerzellen (NK-Zellen) sowie durch die Freisetzung proinflammatorischer Zytokine. Das Zusammenspiel dieser Faktoren bewirkt eine Hemmung regenerativer Prozesse (siehe oben) sowie genomische Instabilität und das verstärkte Auftreten genomischer Mutationen [65–67].

Im Falle der chronischen HCV-Infektion kommt es zu einer milderen Entzündung, deren Pathologie auch deutlich von weiteren Faktoren wie Alter, Koinfektionen, Geschlecht und Alkoholkonsum abhängt. Die HCV-Infektion triggert Antworten von Interferon Typ I und III und somit die Expression einer Reihe von interferonstimulierten Genen (ISGs). Gleichzeitig kommt es zu HCV-spezifischen CD8+/CD4+-T-Zellantworten und zur Aktivierung von NK-Zellen, was zur Freisetzung proinflammatorischer Zytokine führt und zu einem deutlichen Anstieg des Spiegels von ROS. Dies wiederum bedingt u. a. Lipidperoxidation, mitochondriale Dysfunktionen, die ggf. zu einer weiteren Erhöhung des ROS-Spiegel beitragen können und zu DNA-Schäden. Es ist hierbei zu diskutieren, inwieweit ein erhöhter ROS-Spiegel auch durch Modifikation des RNA-Genoms zur genetischen Variabilität von HCV und somit zur Entstehung von *Escape-*Mutanten beitragen könnte. Das Zusammenspiel zwischen ineffizienter Destruktion HCV-positiver Hepatozyten, permanenter Inflammation und unvollständiger Regeneration ist ursächlich für die Zirrhoseentwicklung und erfolgt oft über einen Zeitraum von mehr als 20 Jahren in ca. 10–20 % der Patienten mit einer chronischen HCV-Infektion [36, 58].

## Ausblick

Trotz großer Fortschritte in der Charakterisierung der viralen Lebenszyklen und der Entwicklung robuster antiviraler Strategien bleibt eine Vielzahl von Herausforderungen bestehen. Dazu zählen ein besseres Verständnis der Mechanismen, die zur Entwicklung der virusassoziierten Pathogenese beitragen, sowie insbesondere auch die Relevanz verschiedener Genotypen für Unterschiede in der Pathogenese. Eine wesentliche Herausforderung darüber hinaus ist bei der chronischen HBV-Erkrankung die Eliminierung der cccDNA und im Falle von HCV die Entwicklung und Zulassung einer präventiven und ggf. auch therapeutischen Vakzine.

## Abkürzungen

AKT: Proteinkinase B
ARE: Antioxidant Response Element
BAD: Bcl-2-Antagonist of Cell Death (proapoptotisches Protein)
CBP: CREB-binding Protein (CREB: cAMP Response Element-binding Protein)
CD: Cluster of Differentiation
CDK: Cyclin Dependent Kinase
C/EBP: CCAAT/Enhancer-binding Protein
c‑Src: Tyrosinkinase Src (c-Src: Akronym aus cellular und sarcoma)
DGAT: Diacylglycerol-O-Acyltransferasen
DMV: Double Membrane Vesicle
DNMT: DNA-Methyltransferase
E2F: Elongation Factor 2F
EGFR: Epidermal Growth Factor Receptor
ER: endoplasmatisches Retikulum
FAPP2: Phosphatidylinositol 4‑Phosphate Adaptor Protein 2
GLI2: Family Zinc Finger Protein 2
GSK‑3: Glykogensynthase-Kinase 3
HBeAg: Hepatitis B Virus Early Antigen
HBsAg: Hepatitis B Virus Surface Antigen
HBV: Hepatitis-B-Virus
HBx: Hepatitis-B-X-Protein
HCC: Hepatocellular Carcinoma
HCV: Hepatitis-C-Virus
IRS: Insulinrezeptorsubstrat
JNK: Jun-Kinase
Keap‑1: Kelch-like Ech-associated Protein 1
LHBs: Large Hepatitis B Virus Surface Protein
MAPK: Mitogen Activated Protein Kinase
MHBs: Medium Hepatitis B Virus Surface Protein
MTOR: Mechanistic Target of Rapamycin
NLS: Nuclear Localization Signal
Nrf2: Nuclear Factor Erythroid 2‑related Factor 2
NS: Nichtstrukturprotein
Oct‑1: Octamer-binding Transcription Factor
PTEN: Phosphatase and Tensin Homolog
PI3K: Phosphoinositid-3-Kinase
PI4K: Phosphatidylinositol-4-Kinase
PI4P: Phosphatidylinositol-4-Phosphat
PPARα: Peroxisome Proliferator-activated Receptor Alpha
PRB: Retinoblastom-Protein
PRC2: Polycomb Repressive Complex 2
PTEN: Phosphatase and Tensin Homolog
ROS: Reactive Oxygen Species
SHBs: Small Hepatitis B Virus Surface Protein
SMAD: Mothers against Decapentaplegic Homolog
Smc5/6: Structural Maintenance of Chromosomes Protein 5/6
SREBP‑1: Sterol Regulatory Element-binding Protein
STAT: Signal Transducer and Activator of Transcription
T2DM: Diabetes Typ 2
TERT: Telomerase Reverse Transcriptase
TGFβ: Transforming Growth Factor Beta
